# A one-year exploratory randomized, controlled, multicentric pilot study evaluating the efficacy and tolerance of an anti-hair loss serum containing *Silybum marianum* extract, manganese PCA, and *Lespedeza capitata* extract following hair transplantation in men with androgenic alopecia

**DOI:** 10.3389/fmed.2026.1740785

**Published:** 2026-06-10

**Authors:** Daniel Ortega Quijano, Alba Gómez Zubiaur, Marine Babin, Christophe Lauze, Mélanie Froliger, Sergio Vaño-Galvan, Valérie Mengeaud

**Affiliations:** 1 Disorders Unit of the Ramón y Cajal University Hospital, Hair Disorders and Hair Transplant Unit of Pedro Jaén Group Clinic, University of Alcalá, Madrid, Spain; 2 Trichology Unit, Ricart Medical Institute, Madrid, Spain; 3 Pierre Fabre Dermo-Cosmétique, Toulouse, France; 4 Medical Department, Laboratoires Dermatologiques DUCRAY, Lavaur, France

**Keywords:** androgenetic alopecia, anti-hair loss serum, hair growth, hair loss, hair transplant, plant extract

## Abstract

**Introduction:**

Hair transplantation is a therapeutic option for androgenic alopecia (AGA) with visible results after 1 year. Post-transplant care may aid graft survival and growth, though few studies have assessed its efficacy. We evaluated the long-term tolerance and clinical efficacy of a hair serum containing two plant-derived active extracts (*Silybum marianum*, *Lespedeza capitata*) and manganese PCA, in men undergoing AGA post-hair transplant.

**Methods:**

In this 1-year exploratory randomized controlled trial, hair transplant patients received either study serum with neutral shampoo or shampoo alone. Tolerance and efficacy were evaluated by both investigators and patients at 0.5 month (M0.5) then M1, M3, M6, M9 and M12, depending on parameters, compared to baseline (before hair transplant).

**Results:**

Thirty men [median age: 40.0 (26.0–64.0) years], 15 per group, were included. Daily use of the serum on the recipient area post hair-transplant in men with AGA improved scalp tightness at M1 and discomfort perceived as painful at M0.5 (*p* = NS) and M1 (*p* < 0.05 vs. controls), as well as hair condition at M1 evaluated by patients (*p* < 0.05). The global aspect of the scalp from M1 to M12 and hair growth from M6 to M12 evaluated by investigators showed more pronounced and rapid improvement vs. controls (*p* < 0.05). Serum tolerance and acceptability by patients were excellent.

**Discussion:**

This first randomized controlled pilot trial suggested that this serum enhances global scalp aspect and hair state and appears to promote hair growth after transplantation, thus acting as a safe, dermocosmetic adjuvant for optimal long-term post-procedure hair care.

## Introduction

Alopecia results in a reduction in visible hair, which can have significant psychosocial effects and impair quality of life (1). There are many types of alopecia, but androgenic alopecia (AGA), which is characterized by progressive hair loss, usually in a clearly distributed pattern, is the most common form amenable to surgical treatment (2).

Of the plethora of products available to promote hair growth on the scalp, only two have been approved by the Food and Drug Administration (FDA): finasteride (systemic) and minoxidil (topical) (3). Where conventional treatments have proved disappointing, hair transplant surgery may be considered, mostly by means of follicular unit transplantation or follicular unit extraction techniques (4). Follicular unit transplantation requires the excision of a strip of tissue from the occipital donor area, resulting in linear scarring. Conversely, Follicular unit extraction is a sutureless technique that involves harvesting small individual follicular units from the back of the head using special micropunches, and it can be used to overcome scarring and other follicular unit transplantation complications. The method of implantation in the recipient area is the same for both techniques.

Although performed on an outpatient basis, hair transplantation is an invasive technique, with the following immediate side effects, which typically can last up to 2 weeks: pain, pruritus, inflammation, crusts, erythema and edema. Social exclusion for 15 days after grafting may thus be necessary in some cases. A second period of inflammation may occur 1 month after transplantation and usually lasts up to 3 months. Apparent growth of transplanted hair begins from the 6th month but the anticipated result is really only achieved after 1 year (5, 6).

An international consensus has proposed the administration of specific shampoos and lotions to reduce anti-inflammatory activity in the post-transplantation period, and to improve overall results, and has also proposed that further scientific research is needed to confirm potential benefits (7). A product that improves graft survival, promotes anchoring, reduces inflammation associated with ‘red scalp syndrome’ (8), aids in anagen hair follicle repair, and stimulates the hair cycle and anagen phase could form an integral to post-transplant care. To date, only a few published studies have assessed the efficacy of products applied after hair transplantation, apart from a study evaluating a mild shampoo (9). We have previously shown that an a anti-hair loss serum containing two plant-based active ingredients - a patented *Silybum marianum* extract (WO/2021/023820), and a patented *Lespedeza capitata* extract (WO/2020/020791A1) – and manganese PCA, has a stimulating effect on hair growth *in vitro* (10) and has proven its efficacy against chronic hair loss in a controlled clinical trial (11).

The aim of this randomized, controlled exploratory pilot trial was to generate first results regarding the tolerance and efficacy of a new topical anti-hair loss serum used as a post-transplant adjuvant product on hair and scalp for the first 12 months after hair transplant compared to a control group that received only a neutral shampoo, which was also used by the test group.

## Materials and methods

### Study design

This exploratory multicentric, open-label, randomized, parallel-group, controlled study (No. NCT06576492) was conducted between July 2021 and June 2023 in male subjects undergoing hair transplant for AGA in three centers in Spain (2 in Madrid, 1 in Valencia).

The study was designed to generate first results on efficacy and tolerance rather than to formally test hypotheses. While sharing certain characteristics with pilot studies, its primary objective was not to assess feasibility but to explore clinical signals and inform the design of future confirmatory trials. In this context, and given the absence of prior data in the literature, the sample size was determined based on practical considerations rather than a formal statistical calculation. This approach is consistent with methodological recommendations for exploratory and pilot studies (12–14). In line with these recommendations, our study included 15 subjects per group, which is consistent with commonly accepted sample sizes for exploratory trials (15).

This study was performed in accordance with the Declaration of Helsinki and the spirit of Good Clinical Practice Guidelines (EMA/CHMP/ICH/135/1995), as well as Regulation 2016/679/EU of the European Parliament and of the Council of 27 April 2016 on the protection of individuals with regard to the processing of personal data and the free movement of such data (GDPR) and Spanish regulations.

In accordance with Spanish regulations, the protocol was submitted to an ethics committee (Comité de Ética de la Investigación del Hospital Universitario Ramón y Cajal) before the start of the study and was approved 9 June 2021 under number 030–21.

All study procedures other than hair transplant were non-invasive and painless, and all subjects provided written informed consent before enrolment, after receiving verbal and written information about the study.

### Participants

Men aged ≥18 years, with skin phototypes I to V (Fitzpatrick classification) and requiring hair transplant with micrografts for their AGA, were eligible.

Subjects having any other hair disorder or hair disease (e.g., telogen effluvium, alopecia areata, cicatricial alopecia, hair shaft disorder, trichotillomania), dermatological disorders or ongoing skin lesions on the scalp (e.g., psoriasis, seborrheic dermatitis, severe erythema, severe excoriation, severe sunburn) or any other dermatological condition, and acute, chronic or progressive disease or history of any disease liable to interfere with the study assessments were considered ineligible, as were those having iron deficiency or thyroid disorders (confirmed by laboratory within the previous 3 months).

Subjects with a history of allergy or intolerance to any ingredients of the study product were also excluded, as were those who had undergone radiotherapy or chemotherapy at any time, and those using another local or systemic anti-hair loss treatment or product and antithyroid or iron supplement, in particular any treatment initiated or modified within the previous 3 months (local anti-hair loss treatment and antithyroid or iron supplement) or 6 months (systemic anti-hair loss treatment) before inclusion or planned during the study, or any local or systemic treatment established or modified within the previous weeks and liable to interfere with the study assessments.

The use of concomitant treatments or interventions deemed likely to interfere with the study results was prohibited between the last shampoo and the inclusion visit, and throughout the study.

Subjects were required not to touch or attempt to remove any crusts during the first 48 h after hair transplant, not to cut their hair for 1 month, and had to avoid any exposure of the scalp to UV radiation without protection.

### Products

The test product (Neoptide Expert® anti-hair loss and growth serum, Laboratoires Dermatologiques Ducray®, France) is a serum for the scalp and hair containing a patented *Silybum marianum* extract (*WO/2021/023820*), the manganese salt of L-pyrrolidone carboxylic acid, and a patented *Lespedeza capitata* extract (*WO/2020/020791A1*) as active ingredients (INCI list, Supplementary material). The associated product was a neutral shampoo (Shampooing Extra-Doux®, Ducray Laboratories).

### Randomization procedure

The study was randomized in order to avoid any bias in the allocation to one of the two groups: neither the investigator nor the patient could have an influence on the allocation of the product. The subjects were randomized on a consecutive and chronological basis in two groups according to a randomization list: the test group received the serum (test product) and the neutral shampoo, and the control group received the neutral shampoo only.

### Application modalities and compliance

**Figure 1:**
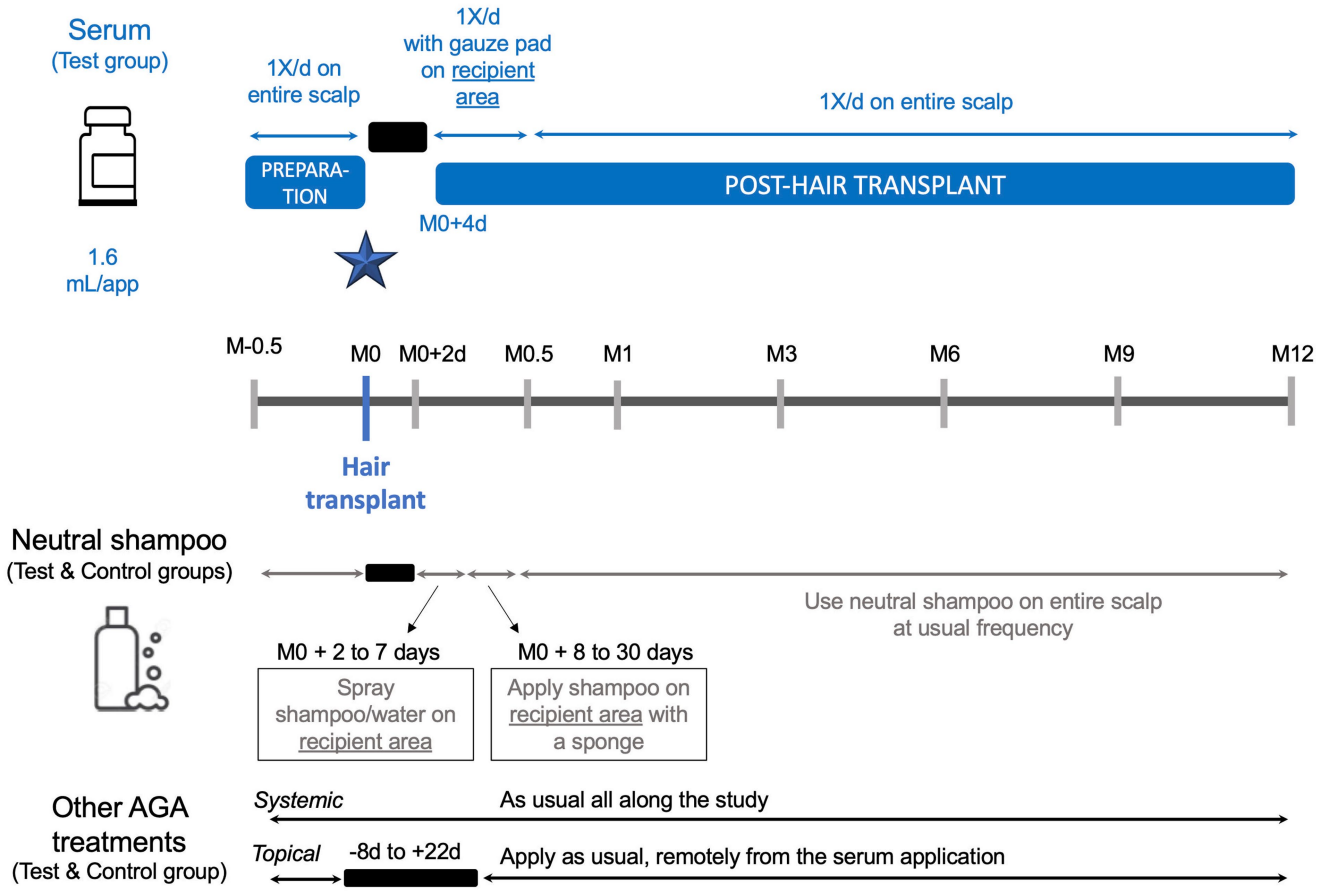
Study design and product application: the schedule of the visits is shown, as well as the conditions of administration of the anti-hair-loss serum containing plant extracts, the neutral shampoo, and other AGA treatments. AGA, Androgenetic alopecia; APP, Application; D, Day.

The product application modalities were explained to each subject at the inclusion visit performed 2 weeks before hair transplant (M-0.5, Figure 1).

Briefly, subjects in the test group were instructed to apply 1.6 mL serum to the scalp (damp or dry) once a day, using a massaging motion with the fingertips to improve product penetration.

The first application of the serum was done during the inclusion visit under the observation of the investigator to evaluate immediate tolerance. Before the hair transplant (during a two-week preparation period), the serum was applied once a day over the entire scalp at home under normal conditions of use. No serum was administered for 4 days after transplantation. From 4 days after hair transplant and until the 15th day (M0.5), subjects were to apply 1.6 mL to a gauze and then apply to the recipient area by light swabbing in order to avoid inadvertent removal of the scabs. The hair was not to be rinsed after application of the serum, and shampoos were prohibited for 9 h following each application of serum. Subjects were asked to record each serum application in their daily log.

All subjects in the test and control groups were instructed to apply the neutral shampoo as follows: (1) before hair transplant, to the entire scalp at the usual frequency, (2) from Day 2 after hair transplant to Day 7 (M0 + 2-7d), subjects were to use a spray containing a mixture of water and shampoo on the micrograft recipient area and let it soak for a few minutes before rinsing thoroughly lukewarm water but with no pressure; (3) from Day 8 to M0.5 (M0 + 8-30d), subjects were to apply the shampoo on the recipient area using a sponge and to gently massage the micrografts to eliminate crusts before rinsing; (4) from M0.5 onwards, subjects could use the shampoo on the entire scalp according to their usual routine, at the subject’s usual frequency.

For each subject completing the study, the theoretical product exposure was around 1 year (maximal product exposure = 56 weeks). Compliance was determined by weighing dials at the beginning and end of the study and by analyzing the subjects’ logs.

### Efficacy evaluation

There were 9 visits over the 12-month study period comprising 6 on-site visits (at inclusion 2 weeks before hair transplant (M-0.5), on the day of hair transplant, before hair transplant (M0), and at M0.5, M6, M9, M12) and 3 teleconsultations (at M0 + 2 to 4 days, M1, and M3). The investigator recorded data in an individual electronic case report form (e-CRF) for each subject, while the subjects recorded self-assessments and clinical scores directly on the paper questionnaires provided.

#### Investigator assessment

Using an internal 5-point Investigator Global Assessment (IGA) scale (−1 = worsening, 0 = stabilization, 1 = slight improvement, less than expected, 2 = moderate improvement, similar as expected, 3 = great improvement, higher than expected), the investigator clinically assessed the recipient area (frontal and/or vertex areas) for the global appearance of the scalp at M0.5, M1, M3, M6, M9, M12, and for hair growth at M6, M9, M12, compared to M0 (immediately prior to hair transplant).

The investigator also assessed physical signs and cosmetic recovery in the scalp recipient area using a numerical rating scale (NRS, ranging from 0 to 10). This included evaluations of erythema and scalp edema at M0 and M0.5, as well as assessments of crusts and healing at M0, M0.5, M1, and M3.

#### Patient assessment

Subjects completed self-assessment questionnaires covering three evaluation criteria:

- Global efficacy on the recipient area, in particular, hair state in the recipient area assessed at M0.5, M1, M3, M6, M9, M12 and monthly between each visit, using an internal 5-point patient global assessment (PGA) scale (−1 = worsening, 0 = stabilization / no change, 1 = slight improvement, 2 = moderate improvement; 3 = great resolution), compared to M-0.5 (inclusion).
- Functional signs in the recipient area, including discomfort perceived as painful, warming sensation, itching, and tightness of the scalp, rated on an NRS (0–10) at each visit from M0 onwards. At M0 + 2 days, the assessment was performed before and after the shampoo.
- Satisfaction, cosmetic acceptability, and hair/scalp perception were assessed at M0, M1, M6 and M12.

### Tolerance evaluation

The investigator evaluated the tolerance of the study products in subjects of both groups by assessing the intensity of physical signs (erythema, oedema, desquamation, dryness, vesicles, papules, seborrhoea, dandruff, dry hair, matted hair) on the entire scalp (before hair transplant) or on the recipient area (after hair transplant), on an NRS (0–10) as follows: 0 = none, 1–3 = mild, 4–7 = moderate, 8–10 = severe at each visit from M-0.5. All physical signs rated as either new or more intense than the previous evaluation on the intensity scale were reported as adverse events (AEs) (except for expected signs related to the hair transplant) and were fully described, including any signs other than those listed.

Global tolerance was assessed by the investigator, both for groups and for individual subjects, at M0 (before transplant) and at M12 (end of study) considering all individual AEs and their characteristics, based on the following 5-point scale: excellent (no functional or physical signs related to the study products observed or reported by subjects); very good; good; moderate; or poor.

Tolerance was also evaluated in terms of functional signs in the recipient area, rated by the patient, including stinging, warming sensation, itching, and tightness of the scalp, on an NRS (0–10) at each visit from M-0.5. It was also assessed using the functional signs rated by the patient before and after application of the serum upon introduction of the serum (M-0.5) and upon reintroduction of the serum after the hair transplant (M0 + 4D).

### Statistical analysis

Due to the exploratory nature of the study, all statistical results were expressed in a descriptive form. All hypothesis tests should be considered exploratory.

Descriptive quantitative variables (demography, self-assessment questionnaires) were presented using numbers, means with standard deviations (SD), medians with minimum and maximum values, or percentages. Qualitative variables were presented using frequencies and percentages.

Both intragroup and intergroup analyses were performed on parameters evaluated in terms of changes from M0 or M-0.5 for PGA only.

For the parameters evaluated on the 5-grade IGA and PGA scales, intragroup analysis was performed after grouping responses of “-1 and 0 “into a class rated (−) and responses of “1, 2 and 3″ into a class rated (+). The sign test was performed at each visit. Intergroup analyses were performed by using the Wilcoxon test between groups at each visit in question.

For erythema and scalp edema scores and analysis of covariance were carried out with the group as the fixed effect and baseline value as the covariate. For crusts and healing scores, analysis of covariance for repeated measures was performed taking the group, the visit and the interaction group visit as fixed effects, the baseline value as the covariate, and subject as the repeated effect. For functional sign scores, analysis of variance for repeated measures was performed. For all physical signs, intergroup comparisons were performed using differences of least-square means.

Statistical analyses were performed using SAS® (version 9.4), and statistical significance was set at <0.05.

## Results

### Participants

Overall, 30 men aged 40.2 ± 9.9 (median 40.0, range 26.0–64.0) years with AGA were included in the study: 15 in the test group and 15 in the control group (Supplementary Figure 1). All patients (100%) underwent follicular unit extraction hair transplantation. No apparent clinical intergroup differences in baseline demographic, hair, or clinical characteristics were observed (Table 1). There were no premature withdrawals and no major deviation or bias, and all subjects (100%) were therefore included in the analysis set.

**Table 1:** Baseline characteristics of the study subjects.

Parameters	Test group (*N* = 15)	Control group (*N* = 15)	Overall (*N* = 30)
Age (years)			
mean ± SD, median [range]	36.4 ± 8.3 *32 [26–57]*	43.9 ± 10.1 *43 [31–64]*	40.2 ± 9.9 *40 [26–64]*
Phototype, *n* (%)			
Phototype II	6 (40.0)	5 (33.3)	11 (36.7)
Phototype III	9 (60.0)	10 (66.7)	19 (63.3)
History of alopecia (years)			
mean ± SD, *median [range]*	11.3 ± 4.6 *11 [4–20]*	17.9 ± 10.8 *13 [1–41]*	14.6 ± 8.8 *12 [1–41]*
Hair transplant technique, *n* (%)			
Follicular unit extraction	15 (100)	15 (100)	30 (100)
Subjects on concomitant AGA treatment^a^, *n* (%)			
Systemic and topical	7 (46.7)	2 (13.3)	9 (30.0)
Systemic only	8 (53.3)	8 (53.3)	16 (53.3)
None	0 (0)	5 (33.3)	5 (16.7)
Concomitant AGA treatments^b^, *n* (%)			
Oral finasteride/dutasteride	13 (86.7)	10 (66.7)	23 (76.7)
Oral minoxidil	12 (80.0)	9 (60.0)	21 (70.0)
Topical minoxidil	7 (46.7)	2 (13.3)	9 (30.0)
Other oral	0 (0)	1 (6.7)	1 (3.3)
Other topical	1 (6.7)	0 (0)	1 (3.3)

AGA, androgenic alopecia; N or n, number of subjects; SD, standard deviation. ^a^Treatment initiated before inclusion and ongoing throughout the study. ^b^A single subject could be undergoing several concomitant treatments.

Compliance with prescribed study product use over the 12-month study period was excellent, with no subjects reporting any omissions or changes in frequency of application (Supplementary Table 3).

### Post-transplant efficacy

#### Investigator assessment

**Figure 2:**
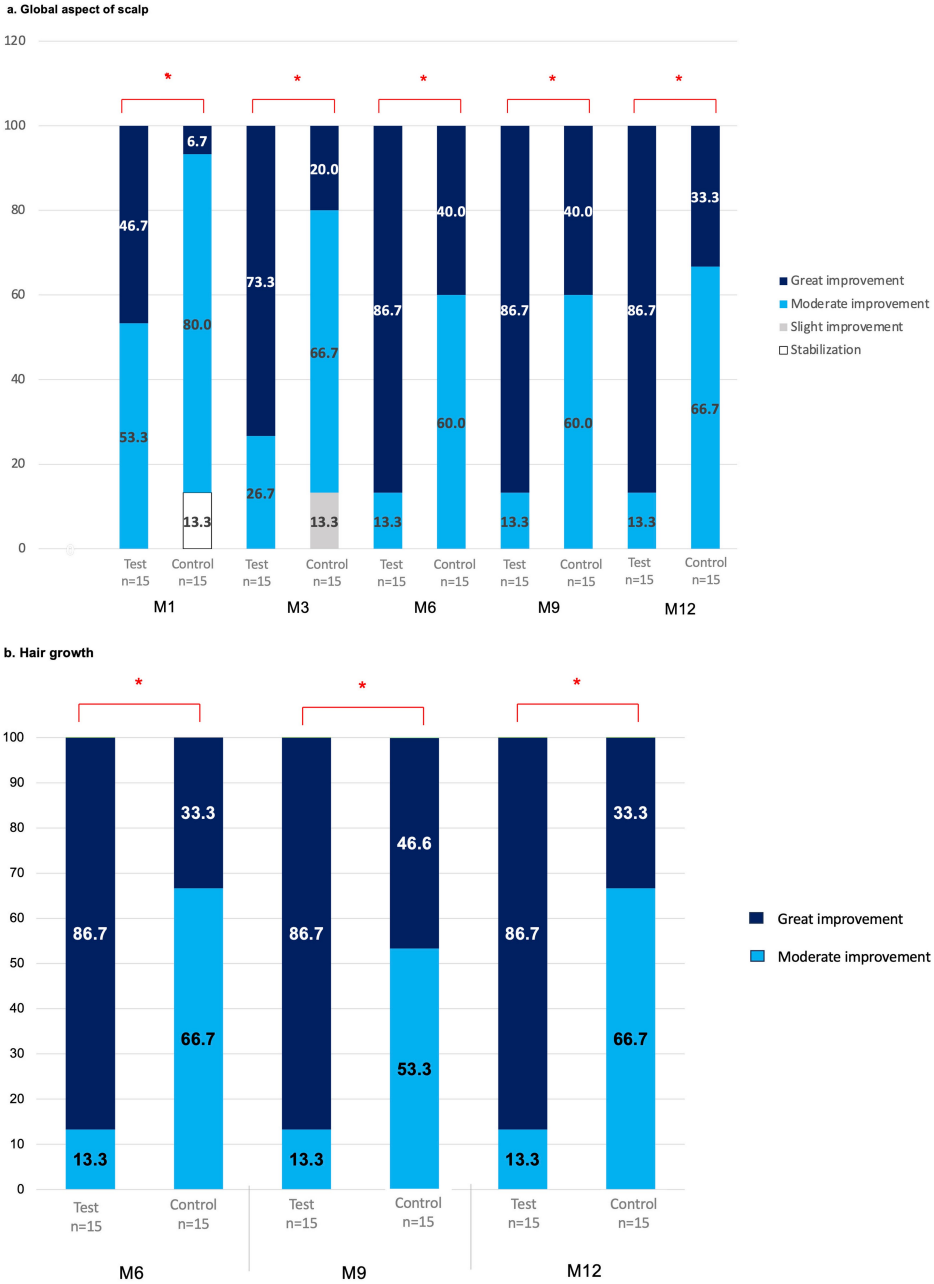
Investigator assessments: **(a)** Global aspect of the scalp, **(b)** Hair growth, *p < 0.05 (intergroup significant difference).

The improvement (considering slight, moderate and great improvement) of the global aspect of the scalp on the recipient area was more marked and rapid in the test group than in the control group between M1 and M12 (*p* < 0.05; Figure 2A). Notably, the percentage of subjects showing great improvement was consistently higher in the test group compared to the control group at each time point (Figure 2A). There were no reported cases of scalp worsening over the entire study period.

Hair growth on the recipient area evaluated on the IGA scale also showed significant differences between the test and control groups at M6, M9 and M12 in favor of the test group (*p* < 0.05; Figure 2B). In particular, a high percentage of subjects with great improvement was found in the test group as early as M6 (86.7%) and remained stable until the study end.

As more subjects in the test group had concomitant AGA treatments than in the control group (100% vs. 67%, Table 1), a complementary analysis was performed excluding the subjects without AGA treatment in the control group (5/15, 33%) for the global aspect of the scalp and hair growth endpoints. These analyses showed no changes in intergroup results compared to the full analysis set.

Scalp soothing at M0.5 was assessed by rating physical signs. Although the intergroup difference in erythema did not reach clinical significance, there was a highly significant increase in erythema in the control group at M0.5 compared to pre hair transplant (M0). In contrast, there was no significant increase in erythema in the test group at M0.5 vs. M0 (Supplementary Table 1).

Crusts were generally present on the recipient area at M0.5 (with maximum scores between 4 and 6 in both groups) before resolving in most subjects at M1 (only 3 subjects had scores between 1 and 4) (Supplementary Table 1; Supplementary Figure 2a). In parallel, scalp healing markedly increased at M0.5 in both groups (scores between 5 and 10 vs. 0 at baseline), before reaching total healing at M1 (all scores between 8 and 10) (Supplementary Table 1; Supplementary Figure 2b). Overall, these results showed good healing on the scalp recipient area within 1 month post-transplant in both groups.

#### Patient assessment

**Figure 3:**
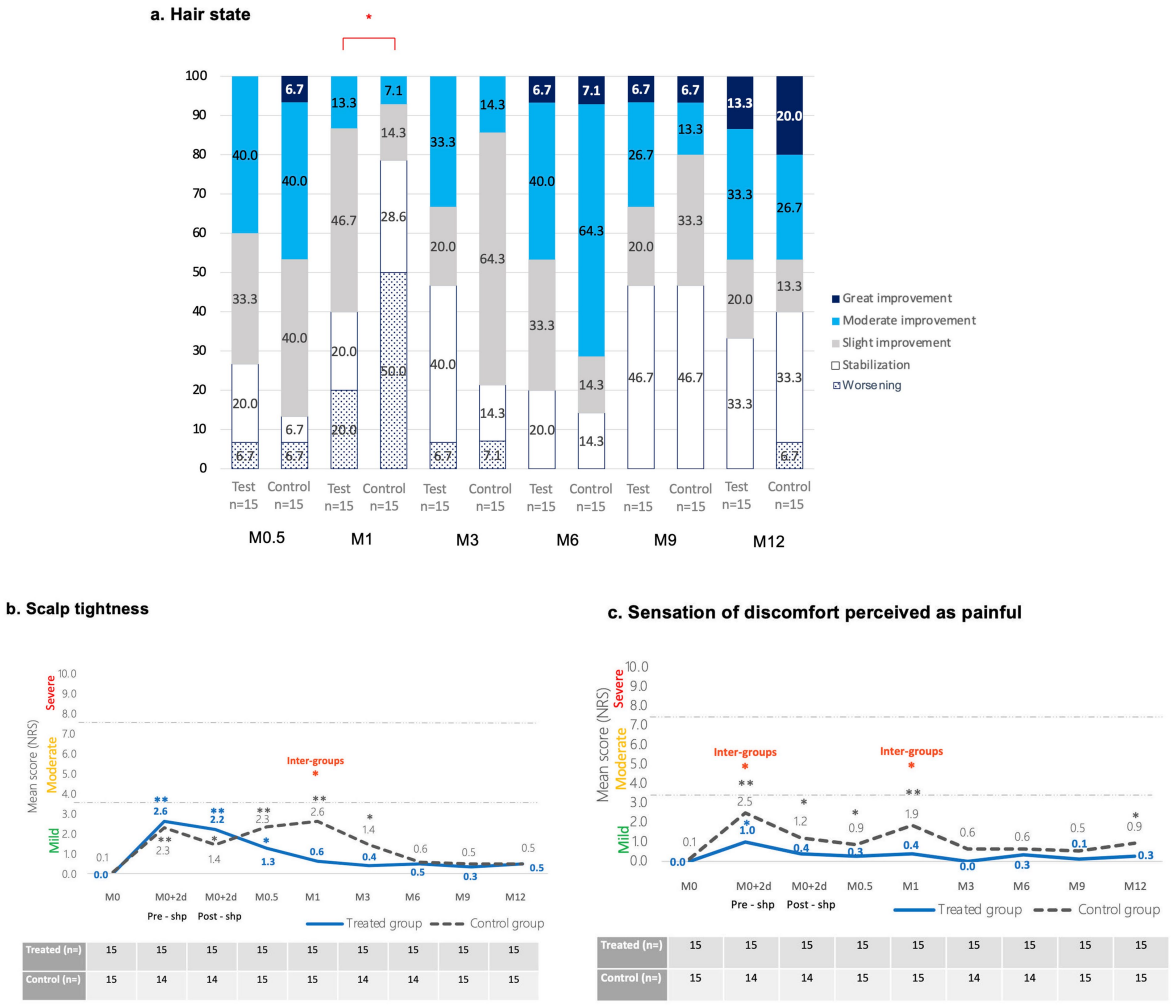
Patient assessments: **(a)** Hair state, **(b)** Scalp tightness, **(c)** Sensation of discomfort perceived as painful, * *p* < 0.05 intragroup (in black) or intergroup (in red), *** *p* < 0.0001. NRS, Numerical rating scale; pre SHP, before shampoo.

Hair state improved more rapidly in the test group 1 month after the transplant, as indicated by the ratings on the PGA scale, which showed a significantly greater percentage of patients who reported improvement (consisting of slight, moderate and great improvement) at M1 in the test group than in the control group (*p* < 0.05; Figure 3A). Global improvement in hair state continued in both groups until M12. At M12, cases of worsening were reported in the control group only (6.7% of patients).

Regarding functional signs during the follow-up period after hair transplant, scalp tightness (Figure 3B) and sensation of discomfort perceived as painful (Figure 3C) decreased significantly more rapidly in the test group (*p* < 0.05 at M1 for both functional signs and at M0 + 2D for sensation of discomfort perceived as painful).

Conversely, there was no significant intergroup difference in the mean scores for itching and warming sensation at any time points (Supplementary Table 2). Subjects also reported great satisfaction with the test product (100% of subjects had an overall satisfaction score ≥5 at M12), reporting effects on hair density (both recipient frontal and donor temporal areas) and hair growth (entire scalp), assessed by greater change from M0 to M12 vs. the control group.

Subjects were also satisfied with the cosmetic effects on hair, with high percentages of subjects with scores ≥5 rating their hair as supple (92.9%), soft (85.7%), easy to style (92.9%), brilliant (71.4%), and having good hold (78.6%) at M12.

### Tolerance

In all, 22 subjects experienced at least one dermatological AE, none of which was serious and with the majority being mild. No patients changed the modality of application and/or temporarily interrupted application of the test product due to a reaction, nor withdrew from the study due to reactions to the test product.

At inclusion (M-0.5), no worsening of the mean intensity of functional signs was reported by patients after the initial serum application (mean NRS on a 10-point scale approximately 0). Following 2 weeks of serum application before hair transplant (M0), there was no worsening of the mean intensity of physical or functional signs in either group (mean NRS on a 10-point scale approximately 0) from inclusion (M-0.5), and global tolerance for the preparation period was rated by the investigator as excellent in 100% of subjects.

From the start of post-transplant serum application (fourth day after transplant), none of the physical signs (e.g., dandruff, desquamation, oedema, dryness) were considered by the investigator as dermatological adverse events suspected as being related to the test product. Some functional signs (itching, tightness, stinging, warming sensations) were reported as dermatological adverse events suspected of being related to the test product, but causality of the related reactions was unlikely, and in any case, not clearly attributable to the test product in the investigator’s opinion. Global tolerance was rated by the investigator as excellent in 100% of subjects.

## Discussion

This exploratory study collected the first data on the tolerance and efficacy of an anti-hair loss serum containing *Silybum marianum* and *Lespedeza capita* extracts and manganese PCA actives on the scalp and hair of men with AGA following hair transplant. Conducted under close dermatological supervision with an extended follow-up period of 1 year, this comparative study demonstrated excellent tolerance for the serum, including on injured skin, as early as 4 days after transplantation and throughout 1 year of daily use. Additionally, it effectively mitigated several adverse effects on the scalp commonly associated with transplant procedure and contributed to improved hair outcomes and hair growth. Notably, compliance among participants was excellent, underscoring the potential value of the serum as a reliable post-transplant adjuvant.

To date, no studies have investigated the long-term effects of adjuvant dermocosmetic products after hair transplantation other than the use of a mild shampoo (9). However, an international expert panel has suggested that dedicated hair care, including specific shampoos and lotions, may help reduce inflammation in the post-transplantation period and improve overall results (7). The panel recommends using products with demonstrated tolerance and efficacy profiles in preclinical and clinical trials (16) as adjuvant therapies, especially taking into account their cosmetic effects. This study is the first to evaluate the efficacy and tolerability of a dermocosmetic serum during the post-transplant period.

In our study, tolerance of the serum was evaluated on compromised skin, as reintroduction of application began just 4 days after hair transplantation. Upon reintroduction of the serum on the fourth day, there was no worsening in the mean intensity of functional signs before and after application of the serum, demonstrating excellent scalp tolerance. Moreover, the serum exhibited a mild soothing effect, as indicated by a significantly earlier reduction in scalp tightness and discomfort, compared with the control group. Notably, discomfort perceived as painful decreased at M0 + 2D and M1 in the test group. In addition, long-term tolerance was assessed throughout 1 year of once-daily use, and global tolerance was rated by the investigator as excellent in 100% of subjects. Compliance with daily use of the study product over the 12-month study period was also excellent.

The neutral shampoo used in our study contained a mild surfactant and was devoid of potentially irritating ingredients, and it has been commercially available in several European countries since March 2011; it is also classified as class I (very good tolerance) for cosmetovigilance (determined in November 2020 for the period August 2019 to July 2020). Indeed, it is highly recommended that gentle but effective shampoo of the scalp be performed once daily to prevent crusting, starting from day 2 post-transplantation (4, 9, 17).

In terms of efficacy of the serum, the effect on hair growth could be assessed from M6, as 6 months is the time required for the transplanted hair follicle to re-enter the hair cycle and begin to grow (5, 6). We demonstrated that one effect of the serum was to help to promote hair growth in the recipient area evaluated on an IGA scale at M6, M9 and M12 (*p* < 0.05). The ability of this serum to improve hair follicle growth had previously been investigated *in vitro* on markers of hair growth and anchorage in human follicle dermal papilla cells and in an *ex vivo* model of human scalp skin (10). This study showed that the active ingredients present in the serum improve anchorage and microcirculation by acting on several signaling pathways (EGFR/PDGFR, Wnt/*β*-catenin and VEGF). This serum has also been clinically tested in the treatment of a form of chronic hair loss in women. Used in more than 60 women with chronic telogen effluvium during 4 months, it demonstrated good tolerance and a significant anti-hair loss effect (11).

Moreover, the improvement in global aspect of the scalp in the recipient area was more marked and rapid in the test group than in the control group between M1 and M12 (*p* < 0.05). Subjects were also satisfied by the cosmetic effect on hair, with high percentages of subjects recording scores ≥ 5 at M12 for hair rated as supple, soft, easy to style, brilliant and with good hold.

However, we acknowledge several limitations in our study. The sample size was relatively small (*n* = 30) because this was an exploratory study aimed at detecting a trend or effect of the product.

However, despite the limited sample size, several statistically significant differences were observed between groups, supporting the relevance of the findings and justifying further investigation in larger studies. This is all the more pertinent given the innovative nature of both the product (including evidence-based ingredients) and the indication. Further experiments could be conducted in larger number of patients, including women, to increase the statistical power and generalizability of our results. Indeed, in our study, the population was made up exclusively of men because the incidence of AGA is much higher in this population. This bias may prevent generalizability of the results. Of note, the test group was numerically younger than the control group. However, this difference was not considered clinically sufficient by the investigators to meaningfully influence post-transplant healing or the interpretation of the study outcomes.

Moreover, in spite of randomization, the two groups showed differences in concomitant AGA treatments, with 100% of subjects using AGA treatments in the test group compared with 67% of subjects in the control group. AGA treatments, prescribed in hair transplant patients to avoid deterioration of the non-transplanted hair, were allowed in the protocol under some strict conditions of standardization and stabilization in line with the recommendations of the international expert consensus statement focusing on pre and post hair transplantation care (7), To address this imbalance, a complementary sensitivity analysis was performed after excluding subjects without AGA treatment in the control group for three main criteria (global aspect of the scalp, hair growth and hair state). This additional analysis, conducted as an exploratory approach, was not used to conclude on the test product effect but only to confirm that the results showed similar intergroup differences than those obtained with the main analysis (with all subjects), supporting the robustness of the findings. It is worth noting that, even for patients who had been receiving concomitant treatments for several months prior to inclusion, the transplant was still necessary. Moreover, the main evaluation criteria were based on intra-subject changes over time. Overall, while residual confounding factor cannot be entirely excluded, the consistency of results across analyses supported the robustness of the findings.

Given that improvement in most physical and functional signs started very early after hair graft, much earlier assessment of these endpoints within the first 2 weeks post-transplant should be considered to reveal potential significant between-group differences.

In addition, further experiments could be conducted with a placebo consisting of basic serum without the three actives. Finally, to date, there is no standardized method for evaluating post-transplant regrowth, so we relied on expert investigators and their current practice for assessment of this parameter.

Nevertheless, the study also has several strengths. First, to our knowledge, this is one of the few studies to have assessed the effectiveness of products applied after hair transplantation, aside from a study that evaluated a mild shampoo (9).

Secondly, the RCT design and the 12-month duration of the study allowed to verify the continued efficacy and tolerance of the product during 1 year, which was in line with the excellent patient compliance throughout the long-term use of the product.

Thirdly, assessment of the serum efficacy was performed by comparing it with a control group that did not apply any care product to the scalp and used the same neutral shampoo as the test group for routine hair care. This parallel-group design ensured reliability of efficacy results, both immediately and later after hair transplant. However, it did not prevent inter-individual variability (Supplementary Figures 2a,b), which may have contributed to some statistically non-significant intergroup differences within this small patient sample size.

In conclusion, this exploratory randomized pilot trial was the first to show that the one-year daily application of the serum to the recipient area following hair transplant in men with AGA significantly improved several post-transplant functional signs, both immediate and late-onset. The global aspect of the scalp, and hair state improved significantly more than in the control group. The serum also appeared to support hair growth. The product demonstrated excellent tolerance and patient acceptability all through the study, indicating that it could be safely recommended as an effective adjuvant dermocosmetic for post-transplant hair care. A larger fully powered trial would be needed to confirm these findings.

## Data Availability

The original contributions presented in the study are included in the article/Supplementary material, further inquiries can be directed to the corresponding author.
