# Supplementary material for: A one-year exploratory randomized, controlled, multicentric pilot study evaluating the efficacy and tolerance of an anti-hair loss serum containing Silybum marianum extract, manganese PCA, and Lespedeza capitata extract following hair transplantation in men with androgenic alopecia
Source: Front Med (Lausanne). 2026 Jun 10;13:1740785. doi: 10.3389/fmed.2026.1740785 (PMC13292112; doi:10.3389/fmed.2026.1740785)
Supplement: Supplementary file 1 [file Supplimentary_file_1.docx]

Supplementary Material

**INCI composition of the study products**

Full International Nomenclature of Cosmetic Ingredients (INCI) list of the anti-hair loss serum (Neoptide Expert®, Laboratoires Dermatologiques Ducray®) used in the study is disclosed below. Ingredients are listed in accordance with cosmetic regulatory labeling requirements (descending order of concentration for ingredients >1%). Exact concentrations of individual ingredients are confidential and cannot be disclosed; however, all key active ingredients investigated in this study are clearly identified in the main manuscript.

Anti-hair loss serum (Neoptide Expert®, Laboratoires Dermatologiques Ducray®:

| N° | Ingredients |
| --- | --- |
| 1 | PURIFIED WATER |
| 2 | CITRIC ACID MONOHYDRATE |
| 3 | SODIUM PHYTATE |
| 4 | ETHYL ALCOHOL 96% |
| 5 | GREEN PENTYLENE GLYCOL |
| 6 | HEXYLENE GLYCOL |
| 7 | SOD. DILAURAMIDOGLU. LYS. 27% |
| 8 | ACRYLATE(POLY)13 MEL |
| 9 | EXT.FL.LESPEDEZA |
| 10 | MILK THISTLE EXTRACT/iPrOH/PEG300 |
| 11 | PYRROLID. CARBOXYL. MANGANESE |

# Supplementary tables and figures

**Supplementary Table 1: Change in physical signs in recipient area assessed by the investigator at each visit from hair transplant (M0), using a numerical rating scale (0 to 10).** *SD: Standard deviation*

|  | | **Randomization group** | | | | | | **P inter-group** |
| --- | --- | --- | --- | --- | --- | --- | --- | --- |
|  | | **Test group (n=15)** | | | **Control group (n=15)** | | |  |
| **Physical Sign** | **VISIT** | **Mean +/- SD** | **Median  [Min-Max]** | **P intra-group  (vs. M0)** | **Mean +/- SD** | **Median  [Min-Max]** | **P intra- group (vs. M0** |  |
| **Crusts** | M0 | **0.00** ± 0.00 | **0.00** [0.00-0.00] | / | **0.00** ± 0.00 | **0.00**[0.00-0.00] | / | / |
|  | M0.5 | **2.60** ± 1.64 | **2.00** [0.00-6.00] | <.0001 | **1.60** ± 1.30 | **2.00**[0.00-4.00] | <.0001 | 0.0055 |
|  | M1 | **0.07** ± 0.26 | **0.00 [**0.00-1.00] | 0.7887 | **0.33** ± 1.05 | **0.00**[0.00-4.00] | 0.1826 | 0.4492 |
|  | M3 | **0.00** ± 0.00 | **0.00** [0.00-0.00] | 1.0000 | **0.00** ± 0.00 | **0.00**[0.00-0.00] | 1.0000 | 1.0000 |
| **Healing** | M0 | **0.00** ± 0.00 | **0.00**[0.00-0.00] | / | **0.00** ± 0.00 | **0.00**[0.00-0.00] | / | / |
|  | M0.5 | **8.20** ± 1.52 | **8.00**[5.00-10.00] | <.0001 | **8.87** ± 1.19 | **9.00**[7.00-10.00] | <.0001 | 0.0291 |
|  | M1 | **9.87** ± 0.52 | **10.00**[8.00-10.00] | <.0001 | **9.93** ± 0.26 | **10.00**[9.00-10.00] | <.0001 | 0.8248 |
|  | M3 | **10.00** ± 0.00 | **10.00** [10.00-10.00] | <.0001 | **10.00** ± 0.00 | **10.00**[10.00-10.00] | <.0001 | 1.0000 |
| **Erythema** | M0 | **0.93** ± 0.96 | **1.00**[0.00-2.00] | / | **0.40** ± 0.83 | **0.00**[0.00-2.00] | / | / |
|  | M0.5 | **1.73** ± 1.03 | **2.00**[0.00-4.00] | 0.0693 | **1.73** ± 1.33 | **1.00**[0.00-5.00] | 0.0002 | 0.1016 |
| **Oedema** | M0 | **0.00** ± 0.00 | **0.00**[0.00-0.00] | / | **0.00** ± 0.00 | **0.00**[0.00-0.00] | / | / |
|  | M0.5 | **0.13** ± 0.52 | **0.00**[0.00-2.00] | 0.3259 | **0.13** ± 0.52 | **0.00**[0.00-2.00] | 0.3259 | 1.0000 |

**Supplementary Table 2: Changes in warming sensation and itching on recipient area assessed by patients at each visit from hair transplant (M0), using a numerical rating scale (0-10).** *SD: Standard deviation*

|  | | **Randomization group** | | | | | | | **P inter-group** |
| --- | --- | --- | --- | --- | --- | --- | --- | --- | --- |
|  | | **Test group (n=15)** | | | **Control group (n given below)** | | | |  |
| **Functional sign** | **VISIT** | **Mean +/- SD** | **Median  [Min-Max]** | **P intra-group (vs. M0)** | **n** | **Mean +/- SD** | **Median  [Min-Max]** | **P intra-group (vs. M0** |  |
| **Itching** | M0 | 0.53 ± 0.92 | 0.00 [0.00-3.00] | / | 15 | 0.20 ± 0.41 | 0.00 [0.00-1.00] | / | / |
|  | M0+2d Before shampoo | 2.33 ± 2.69 | 1.00 [0.00-8.00] | 0.0028 | 14 | 1.79 ± 2.15 | 1.00 [0.00-8.00] | 0.0099 | 0.7764 |
|  | M0+2d After shampoo | 1.73 ± 2.58 | 1.00 [0.00-8.00] | 0.0413 | 14 | 1.29 ± 2.49 | 0.00 [0.00-9.00] | 0.0747 | 0.8693 |
|  | M0+2d Before lotion | 2.33 ± 2.61 | 1.00 [0.00-9.00] | 0.0028 | / | / | / | / | / |
|  | M0+2d After lotion | 2.20 ± 2.54 | 1.00 [0.00-8.00] | 0.0054 | / | / | / | / | / |
|  | M0.5 | 1.80 ± 2.37 | 1.00 [0.00-9.00] | 0.0317 | 15 | 2.27 ± 2.58 | 2.00 [0.00-8.00] | 0.0008 | 0.3448 |
|  | M1 | 1.73 ± 2.25 | 1.00 [0.00-8.00] | 0.0413 | 15 | 2.60 ± 2.44 | 2.00 [0.00-8.00] | 0.0001 | 0.1563 |
|  | M3 | 1.07 ± 1.87 | 0.00 [0.00-7.00] | 0.3547 | 14 | 2.21 ± 2.89 | 1.00 [0.00-9.00] | 0.0018 | 0.1006 |
|  | M6 | 0.93 ± 1.71 | 0.00 [0.00-6.00] | 0.4853 | 14 | 1.29 ± 2.20 | 0.00 [0.00-8.00] | 0.1074 | 0.5068 |
|  | M9 | 0.93 ± 1.62 | 0.00 [0.00-5.00] | 0.4853 | 15 | 1.00 ± 2.14 | 0.00 [0.00-7.00] | 0.1769 | 0.6411 |
|  | M12 | 0.93 ± 1.33 | 0.00 [0.00-4.00] | 0.4853 | 15 | 1.27 ± 2.25 | 0.00 [0.00-7.00] | 0.0729 | 0.4321 |
| **Warming sensation** | M0 | 0.00 ± 0.00 | 0.00 [0.00-0.00] | / | 15 | 0.00 ± 0.00 | 0.00 [0.00-0.00] | / | / |
|  | M0+2d Before shampoo | 0.53 ± 1.25 | 0.00 [0.00-4.00] | 0.1307 | 14 | 1.36 ± 2.10 | 0.00 [0.00-5.00] | 0.0004 | 0.1185 |
|  | M0+2d After shampoo | 0.47 ± 1.25 | 0.00 [0.00-4.00] | 0.1852 | 14 | 1.14 ± 1.99 | 0.00 [0.00-5.00] | 0.0026 | 0.2036 |
|  | M0+2d Before lotion | 0.93 ± 1.91 | 0.00 [0.00-7.00] | 0.0092 | / | / | / | / |  |
|  | M0+2d After lotion | 0.93 ± 1.98 | 0.00 [0.00-7.00] | 0.0092 | / | / | / | / |  |
|  | M0.5 | 0.27 ± 0.80 | 0.00 [0.00-3.00] | 0.4471 | 15 | 0.87 ± 1.55 | 0.00 [0.00-5.00] | 0.0153 | 0.2279 |
|  | M1 | 0.20 ± 0.41 | 0.00 [0.00-1.00] | 0.5683 | 15 | 0.87 ± 1.81 | 0.00 [0.00-7.00] | 0.0153 | 0.1808 |
|  | M3 | 0.00 ± 0.00 | 0.00 [0.00-0.00] | 1.0000 | 14 | 0.43 ± 0.85 | 0.00 [0.00-3.00] | 0.2917 | 0.4511 |
|  | M6 | 0.33 ± 1.05 | 0.00 [0.00-4.00] | 0.3425 | 14 | 0.93 ± 1.54 | 0.00 [0.00-5.00] | 0.0152 | 0.2742 |
|  | M9 | 0.20 ± 0.56 | 0.00 [0.00-2.00] | 0.5683 | 15 | 0.33 ± 0.82 | 0.00 [0.00-3.00] | 0.3425 | 0.7878 |
|  | M12 | 0.40 ± 0.83 | 0.00 [0.00-2.00] | 0.2553 | 15 | 0.27 ± 1.03 | 0.00 [0.00-4.00] | 0.4471 | 0.7878 |

**Supplementary Table 3: Changes in compliance and tubes weights**

| **Change in compliance (No/Yes)** | **Frequency** | **Percent** | **Cumulative Frequency** | **Cumulative Percent** |
| --- | --- | --- | --- | --- |
| **No** | 15 | 100.00 | 15 | 100.00 |

| **Variable** | **N** | **Mean** | **Std Dev** | **Minimum** | **Median** | **Maximum** |
| --- | --- | --- | --- | --- | --- | --- |
| \| **Total weight brought back** \| \| --- \| \| **Total quantity used** \| \| **Average quantity used (per tube)** \| | \| 15 \| \| --- \| \| 15 \| \| 15 \| | \| 1244.89 \| \| --- \| \| 582.37 \| \| 36.06 \| | \| 141.15 \| \| --- \| \| 140.49 \| \| 7.56 \| | \| 887.84 \| \| --- \| \| 291.89 \| \| 20.85 \| | \| 1256.31 \| \| --- \| \| 615.20 \| \| 38.37 \| | \| 1488.93 \| \| --- \| \| 744.41 \| \| 45.43 \| |

On subjects that brought back 17 tubes

| **Variable** | **N** | **Mean** | **Std Dev** | **Minimum** | **Median** | **Maximum** |
| --- | --- | --- | --- | --- | --- | --- |
| \| **Total weight brought back** \| \| --- \| \| \| **Total quantity used** \| \| --- \| \| \| **Average quantity used (per tube)** \| | \| 10 \| \| --- \| \| 10 \| \| 10 \| | \| 1288.51 \| \| --- \| \| 644.90 \| \| 37.94 \| | \| 93.20 \| \| --- \| \| 93.20 \| \| 5.48 \| | \| 1189.00 \| \| --- \| \| 444.48 \| \| 26.15 \| | \| 1268.69 \| \| --- \| \| 664.72 \| \| 39.10 \| | \| 1488.93 \| \| --- \| \| 744.41 \| \| 43.79 \| |

**Supplementary figures**

**Supplementary Figure 1: Flow chart;** *ICF = informed consent form, FAS = full analysis set; PP = Per protocol*


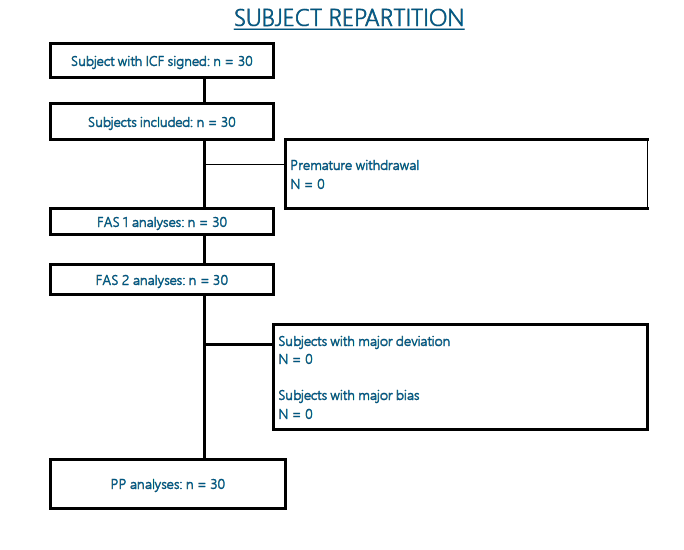


**Supplementary Figure 2: Individual data for change in a) crusts b) healing assessed by the investigator in recipient area, at each visit from hair transplant (M0), using a numerical rating scale (0-10).**

1. **
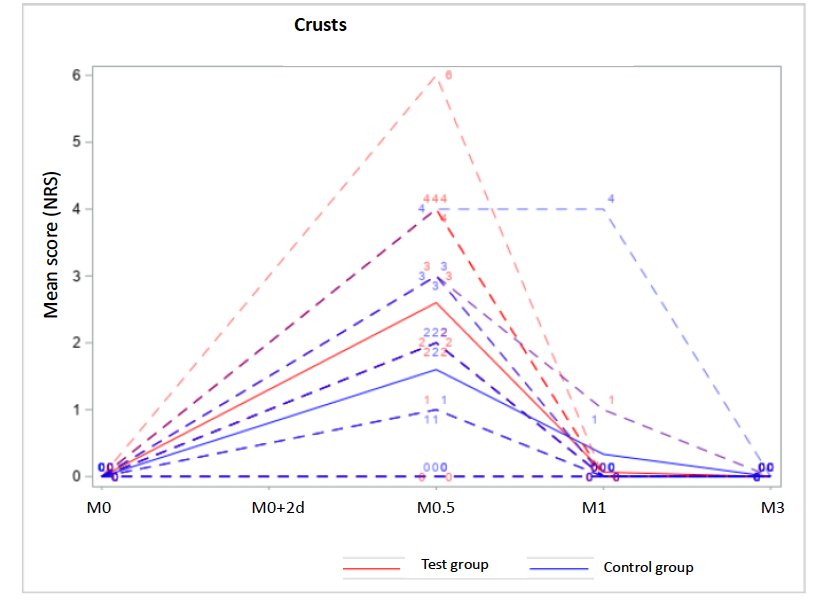
**

**b)** **
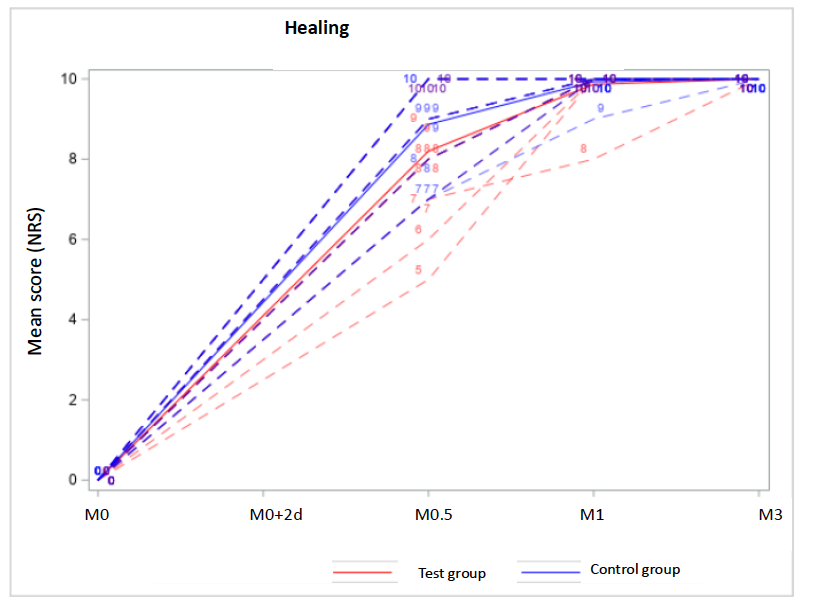
**
